# Extracorporeal Membrane Oxygenation to Support COVID-19 Patients: A Propensity-Matched Cohort Study

**DOI:** 10.1155/2023/5101456

**Published:** 2023-06-12

**Authors:** Björn Stessel, Maayeen Bin Saad, Lotte Ullrick, Laurien Geebelen, Jeroen Lehaen, Philippe Jr Timmermans, Michiel Van Tornout, Ina Callebaut, Jeroen Vandenbrande, Jasperina Dubois

**Affiliations:** ^1^Department of Intensive Care and Anaesthesiology, Jessa Hospital, Hasselt, Belgium; ^2^UHasselt, Faculty of Medicine and Life Sciences, LCRC, Agoralaan, 3590 Diepenbeek, Belgium; ^3^Department of Cardiothoracic Surgery, Jessa Hospital, Hasselt, Belgium; ^4^Department of Cardiology, Jessa Hospital, Hasselt, Belgium

## Abstract

**Background:**

In patients with severe respiratory failure from COVID-19, extracorporeal membrane oxygenation (ECMO) treatment can facilitate lung-protective ventilation and may improve outcome and survival if conventional therapy fails to assure adequate oxygenation and ventilation. We aimed to perform a confirmatory propensity-matched cohort study comparing the impact of ECMO and maximum invasive mechanical ventilation alone (MVA) on mortality and complications in severe COVID-19 pneumonia.

**Materials and Methods:**

All 295 consecutive adult patients with confirmed COVID-19 pneumonia admitted to the intensive care unit (ICU) from March 13^th^, 2020, to July 31^st^, 2021 were included. At admission, all patients were classified into 3 categories: (1) full code including the initiation of ECMO therapy (AAA code), (2) full code excluding ECMO (AA code), and (3) do-not-intubate (A code). For the 271 non-ECMO patients, match eligibility was determined for all patients with the AAA code treated with MVA. Propensity score matching was performed using a logistic regression model including the following variables: gender, P/F ratio, SOFA score at admission, and date of ICU admission. The primary endpoint was ICU mortality.

**Results:**

A total of 24 ECMO patients were propensity matched to an equal number of MVA patients. ICU mortality was significantly higher in the ECMO arm (45.8%) compared with the MVA cohort (16.67%) (OR 4.23 (1.11, 16.17); *p*=0.02). Three-month mortality was 50% with ECMO compared to 16.67% after MVA (OR 5.91 (1.55, 22.58); *p* < 0.01). Applied peak inspiratory pressures (33.42 ± 8.52 vs. 24.74 ± 4.86 mmHg; *p* < 0.01) and maximal PEEP levels (14.47 ± 3.22 vs. 13.52 ± 3.86 mmHg; *p*=0.01) were higher with MVA. ICU length of stay (LOS) and hospital LOS were comparable in both groups.

**Conclusion:**

ECMO therapy may be associated with an up to a three-fold increase in ICU mortality and 3-month mortality compared to MVA despite the facilitation of lung-protective ventilation settings in mechanically ventilated COVID-19 patients. We cannot confirm the positive results of the first propensity-matched cohort study on this topic. This trial is registered with NCT05158816.

## 1. Introduction

Coronavirus disease 2019 (COVID-19) is a viral infection caused by the severe acute respiratory syndrome coronavirus 2 (SARS-CoV-2). A high viral load will cause both a direct viral cytopathic effect as well as an immune response with a cytokine storm, potentially resulting in severe pneumonia and acute respiratory distress syndrome (ARDS) [1]. Treatment of these patients includes intensive care unit (ICU) admission and therapy with conventional methods established for ARDS, including lung-protective mechanical ventilation, neuromuscular blockade, and prone positioning [2].

In the selected patients with severe respiratory failure from COVID-19, treatment with extracorporeal membrane oxygenation (ECMO) can facilitate lung-protective ventilation which may improve outcome and survival if conventional therapy fails to assure adequate oxygenation and ventilation [3–5]. Therefore, International guidelines recommend, depending on the availability of resources, considering venovenous (VV) ECMO in the selected patients with COVID-19 who develop severe ARDS and hypoxemia refractory to prone positioning and optimal ventilator management [2, 6, 7]. More specifically, VV ECMO should be considered in the selected patients with the sustained PaO2/Fi02 ratio (P/F Ratio) < 60 mmHg or pH < 7.20 + PaCO_2_ > 80 mmHg despite maximizing conservative therapies [7]. VA ECMO should be timely considered before the development of multiple organ failure in the selected patients with the coexistence of refractory cardiogenic shock [7]. Besides its known benefits, ECMO support also carries an increased risk of bleeding and thromboembolic events [8] and, therefore, may have a negative impact on the survival rate.

Despite the growing body of literature on ECMO therapy in COVID-19 patients, randomized controlled trials comparing outcomes and adverse events of ECMO therapy versus conventional respiratory support are lacking due to ethical concerns. In the absence of randomized controlled trials (RCTs), propensity-matched cohort studies comparing the outcomes of ECMO therapy versus maximum ventilation alone (MVA) for ECMO-eligible COVID-19 patients in homogeneous cohorts are the best available study design. Different propensity-matched cohort studies has been published with a demonstrated 3-fold improvement in survival with ECMO (75%) compared to MVA (26.2%) [9]. The high survival rate in the ECMO group of this study is not reported in other publications, and as result, confirmatory studies are necessary. Others show an absolute mortality reduction of 18.2% (44% vs. 25.8%) for treatment with ECMO compared to MVA [5].

JESSA hospital, Hasselt, was situated at the epicenter of the Belgian outbreak during the first COVID wave with the highest incidence across the country [10]. From the first admission to ICU on March 13^th^, admissions of critically ill COVID-19 patients to ICU grew exponentially [11] which resulted in very high thresholds for initiating ECMO during the first wave. Indeed, surge conditions result in decreased utilization of EMCO, as constrained resources must be utilized efficiently to ensure an acceptable level of care in all patients [12]. This high threshold for ECMO during the first wave, however, may enhance the selection of a well-matched cohort of COVID-19 patients treated with MVA.

Hence, this study aimed to perform a confirmatory propensity-matched cohort study comparing the impact of ECMO and MVA on mortality and complications in severe COVID-19 pneumonia. The main hypothesis was that the initiation of ECMO therapy in selected patients would reduce mortality and improve clinical outcomes in critically ill COVID-19 patients admitted to ICU.

## 2. Materials and Methods

This single-center, longitudinal, retrospective, investigator-initiated, propensity-matched cohort study was performed at Jessa Hospital, Hasselt, Belgium. This study is approved by the Ethical Committee of Jessa Hospital, Hasselt, Belgium, on 8^th^ September, 2021, and registered on clinicaltrials.gov. (NCT05158816). The requirement for informed consent from the study subjects was waived by the Ethical Committee of Jessa Hospital due to the urgent need to collect data on the ongoing pandemic and the retrospective nature of this study. This study was performed in accordance with all relevant guidelines and regulations and in accordance with the Declaration of Helsinki. The study is reported according to the STrengthening the Reporting of OBservational studies in Epidemiology (STROBE) guidelines [13].

### 2.1. Study Population

All adults (>18 years) with acute hypoxemic respiratory failure due to diagnosed COVID-19 pneumonia and admitted to ICU from 13th March 2020 until 30th June 2021 were included in this analysis. Following the World Health Organisation (WHO) protocol [14], laboratory confirmation of COVID-19 infection was defined as a positive result on polymerase chain reaction (PCR) assays of nasopharyngeal swab samples or bronchoalveolar lavage. Only laboratory-confirmed patients were included in the analysis. Data from 295 consecutive patients admitted to the ICU from March 13^th^, 2020, until July 31^st^, 2021, were prospectively entered into a customized database that included medical history, demographic data, clinical symptoms and signs, laboratory results, ventilator settings, ventilator-derived parameters, and clinical outcomes [15]. This database was retrospectively reviewed [15]. APACHE II and APACHE IV scores were calculated on ICU admission [16, 17]. The sequential organ failure assessment (SOFA) score [18] was evaluated on a daily basis.

All patients were classified into 3 categories on admission based on their medical history, age, and clinical frailty index: (1) full code including potential initiation of ECMO therapy (AAA code), (2) full code excluding ECMO (AA code), and (3) do-not-intubate (A code). Inclusion criteria for ECMO candidacy during the first wave were as follows: prone ventilation, neuromuscular blockade, age <60 years, sustained severe hypoxemia (P/F-ratio <60 mmHg) or hypercapnia (pH < 7.20 + PaCO2 > 80 mmHg) despite maximum ventilator support, and clinical frailty scale of 1 or 2 [15]. After the first wave in May 2020, inclusion criteria were extended to clinical frailty scale <5 and age <70 years (with age between 70 and 80 years only a relative contraindication) [15]. Exclusion criteria for ECMO were known active malignancy, severe chronic organ failure (i.e. hepatic cirrhosis Child-Pugh B or C or COPD GOLD IV), signs of acute, cardiac arrest, severe bleeding, and known severe neurological injury or cognitive impairment (including stroke or dementia) or multiple organ failure involving three or more organ systems [15].

### 2.2. Standard Treatment Procedure [15]

All COVID-19 patients were treated according to the COVID protocol of the JESSA hospital based on the latest insights on COVID-19 at that timepoint [2, 15, 19]. According to this protocol, all patients admitted to our ICU received an intravenous (IV) infusion with glucose 5% at 60 ml/h as maintenance fluid and stress ulcer prophylaxis with pantoprazole 40 mg intravenously daily. Prophylactic antibiotic therapy was initiated for 5 days, using amoxicillin-clavulanic acid 1 g IV 4 q.i.d. or moxifloxacin 400 mg IV QD in case of known allergy to penicillin. Prophylactic administration of antibiotics was abandoned on 08^th^ April 2020. Initially, corticosteroids were administered with caution and minimally after 1 week of ICU admission based on the clinical judgment of the attending intensivist. After the publication of the first results of the RECOVERY trial in July 2020, all patients received intravenous dexamethasone at a dose of 6 mg once daily for ten days after admission. Ventilatory support was initiated with a high-flow nasal cannula or noninvasive mechanical ventilation as long as the patient was cooperative. Awake-prone positioning was also applied in cooperative patients who required support with a high-flow nasal cannula. In case of respiratory fatigue, patients were sedated and intubated and invasive mechanical ventilation (IMV) was started according to the ARDS network guidelines. This was based on the first reports that viral pneumonia caused by SARS-CoV-2 mimicked an ARDS-like pattern [2]. Sedation was performed by a combination of propofol, midazolam, and piritramide in selected cases in association with ketamine, clonidine, or dexmedetomidine, always aiming for the lowest level of sedation required to tolerate IMV. The intermittent use of neuromuscular blocking agents was applied when required. Adjustments were made guided by pulse oximetry levels, which were continuously monitored, and arterial blood gasses took every 4 hours. In case of hypotension due to vasoplegia, norepinephrine was used as the first choice vasopressor.

### 2.3. Anticoagulation

Between March 13th, 2020, and March 30th, 2020, all patients received routine low dose pharmacological VTE prophylaxis, i.e., QD subcutaneous injection of nadroparin calcium 2850 IU. On March 30^th^, 2020, a high incidence of deep venous thrombosis was discovered [20], for which we changed our prophylactic anticoagulation protocol from prophylactic to intermediate dosages of low molecular weight heparin (LMWH) with plasma anti-Xa activity monitoring [11]. The anti-Xa activity was measured daily and targeted at 0.3 to 0.5 IU/ml in patients without echographic findings of deep venous thrombosis (DVT) and 0.4 to 1 IU/ml in patients with screening duplex positive for DVT. Patients were routinely screened for DVT, using ultrasonography twice per week.

Other haemostasis parameters were also measured daily and included the activated partial thromboplastin time (aPTT), international normalized ratio (INR), platelet count, and fibrinogen.

At the initiation of ECMO therapy, LMWH therapy was stopped and unfractionated heparin (UFH) was started. In these patients, aPTT was measured six times per day and targeted at 60–80 seconds in patients without clot formation in the ECMO circuit or echographic findings of DVT and 80–100 seconds in patients with documented thrombus formation.

### 2.4. ECMO Approach [15]

A standard ECMO/ECLS circuit was used for all patients, including a Hico Variotherm 550 heater/cooler, Sechrist gas blender, a LivaNova Stöckert console with a Revolution centrifugal pump system, and a Medtronic Biotrend SvO2 meter. The disposables consisted of a LivaNova Revolution centrifugal pump head with line reassure control in 3 places: P1 negative drainage pressure, P2 preoxygenator pressure, and P3 postoxygenator pressure and a coated VA-tubing set with a PMP fiber ECMO oxygenator (LivaNova EOS ECMO or Eurosets A.L. ONE ECMO). 5000 IU of UFH were administered IV before cannulation according to our protocol. A cardiac surgeon performed the venous drainage cannulation. After disinfection and preparing the groin, a 21Fr. or 25Fr. Medtronic multistage venous cannula was inserted percutaneously with Seldinger technique into the RFV (right femoral vein) under ultrasound guidance. The tip of the cannula was placed into the VCI to avoid recirculation. Simultaneously, the venous return cannula (Edwards Optisite 20Fr. or 22Fr.) was inserted by a cardiac anaesthesiologist into the RIJV (right internal jugular vein) with the tip positioned towards the tricuspid valve. After ultrasound control of the position of both cannulas and ACT check, ECMO was initiated. Blood flow was increased with a target of 2,4 LPM CI (cardiac index), taking the limitations of negative venous drainage pressures into account. Fine tuning of ECMO ventilation/oxygenation settings was performed led by arterial blood gas sampling. A rather high level of PEEP (>10 cm H_2_O) was maintained during ECMO. The pressure-controlled mode of ventilation was preferred with an RR 10–12/min, FiO_2_ tapered to 0.4, a tidal volume target of 4–6 ml/kg, PIP <30 cm H_2_O, and plateau pressures <25 cm H_2_O.

### 2.5. Outcome Parameter

The primary endpoint is ICU mortality indicating the study population was divided into patients who died at the ICU and patients who were discharged from the ICU. The key secondary outcome in this study is 3-month mortality. Other secondary outcomes include the incidence of acute kidney injury and continuous renal replacement therapy (CRRT), other complications during ECMO, length of stay (LOS) in the ICU, and hospital LOS. All patients were followed for at least 3 months after submission to ICU. The data set was closed on October 31st, 2021, ensuring that all patients reached the primary and key secondary outcomes.

### 2.6. ICU Scoring Systems

APACHE II, APACHE IV, and SOFA scores were calculated https://www.via.mdcalc.com within the first 24 hours after admission to our ICU. The data with the highest severity were used to calculate these scores [19].

### 2.7. Definitions

Acute kidney failure was diagnosed according to the KDIGO clinical practice guidelines [21]. ARDS was diagnosed according to the Berlin definition [22]. Sepsis and septic shock were defined according to the 2016 Third International Consensus Definition for Sepsis and Septic Shock.

### 2.8. Statistical Analysis

For descriptive purposes, continuous data are shown as mean ± standard deviation (SD) and categorical data are presented as frequencies (%). For the 271 non-ECMO patients, match eligibility was determined based on the following applied criteria: patients with AAA-code (ECMO candidacy) on admission and treated with IMV. Subsequently, propensity score matching was performed using a logistic regression model including the following variables: gender, P/F ratio, SOFA score at admission, and date of ICU admission. More specifically, the worst P/F-ratio during IMV therapy in the non-ECMO group was compared with the P/F-ratio before starting ECMO in the ECMO group. To minimize the risk of selecting a falsely reduced P/F ratio due to sputum plugs or other mechanical problems, the worst P/F ratio was only selected taking into account the global evolution of P/F ratios over time. “Date of ICU admission” or “wave” was included in the propensity score model to prevent an asymmetrical distribution of patients across groups over time in an attempt to match groups for evolving treatment strategies and different virus variants. The date of ICU admission was categorized according to the COVID-19 wave (supplementary table 1). Waves 1 and 2 in Belgium were caused by the D614G variant, wave 3 by the alpha variant, and wave 4 by the delta variant. These virus variants differ in disease severity and consequently the mortality rate. Furthermore, in the course of the pandemic, we also adapted therapy strategy to several domains. After nearest neighbour calliper matching with a calliper of 0.2 [23], 24 patients without ECMO treatment acted as the matched control group. Comparisons between the groups were performed with the Student's *t*-tests for normally distributed data and with Mann–Whitney *U*-test for not normally distributed data. Categorical variables were analyzed with a Chi-Square test or, if appropriate, with Fisher's exact test. A *p* value <0.05 was considered statistically significant. All analyses were performed with SPPS version 27.

## 3. Results

**Figure 1:**
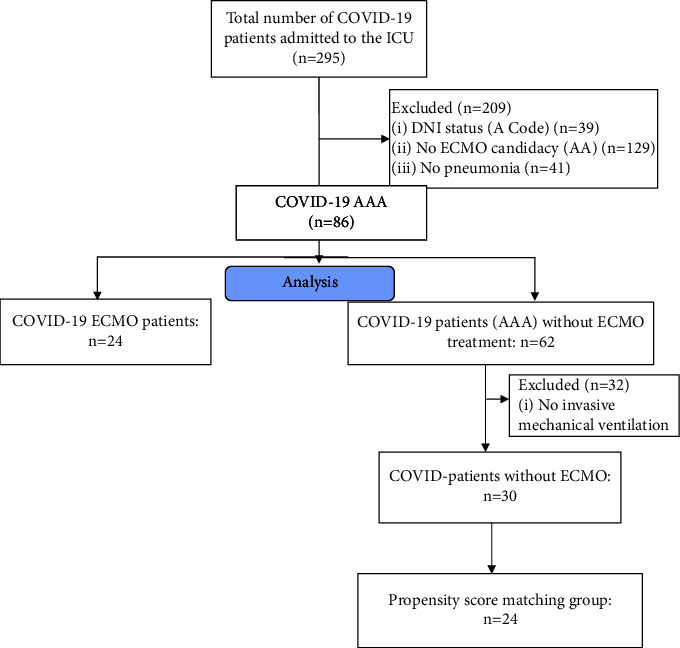
STROBE flowchart depicting inclusion and exclusion.

STROBE flowchart depicting inclusion and exclusion is presented in Figure 1. A total of 295 patients were admitted to the ICU between March, 13^th^, 2020, and October 17th, 2021. 209 patients were excluded for further analysis: 41 patients were admitted for other reasons at the ICU, 39 patients had a do-Not-Intubate (DNI) code and 129 patients were excluded due to no ECMO candidacy. Of the remaining 86 patients, 24 (27.90%) were treated with ECMO, whereas 62 (72.10%) patients did not receive ECMO treatment. After the exclusion of patients without invasive mechanical ventilation, 24 patients were identified as the propensity score matching group. Supplementary information on the distribution of patients across groups stratified for the date of inclusion is presented in supplementary table 1. All patients in the matched cohort group suffered from sustained P/F ratio <60 mmHg or pH < 7.20 + PaCO2 > 80 mmHg during IMV therapy.

Baseline characteristics of the COVID-19 ECMO patients, non-ECMO patients, and the matched cohort are presented in Table 1. No significant differences were found in age, gender, BMI, clinical frailty index, and comorbidities between the groups. Baseline characteristics of the excluded COVID-19 AA patients are presented in supplementary table 2.

ICU mortality in the COVID-19 ECMO group was 45.8% which is significantly higher compared to the non-ECMO patients with an ICU mortality of 6.50% (*p* < 0.001) and compared to the matched cohort with an ICU mortality of 16.67% (*p*=0.02) (Table 2). ICU mortality in the COVID-19 AA group was 28.7%. ICU mortality in the mechanically ventilated AA subcohort was 41.4% (supplementary table 3). A detailed description of complications and causes of death of all COVID-19 ECMO patients is presented in Supplementary Table 4.

**Figure 2:**
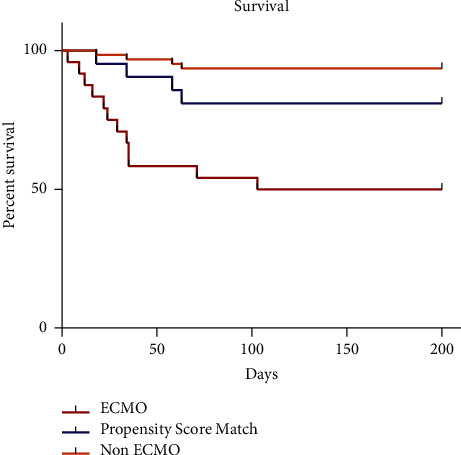
Survival analysis. The Kaplan–Meier survival chart.

The odds ratio for ICU mortality in the ECMO group versus the non-ECMO patients is 12.27 (3.37, 44.69) and versus the matched cohort is 4.23 (1.11, 16.17). The odds ratio for 3-month mortality in the COVID-19 ECMO group compared to the non-ECMO group is 14.50 (3.99, 58.73) and compared to the matched cohort is 5.91 (1.55, 22.58). A survival chart is shown in Figure 2.

**Figure 3:**
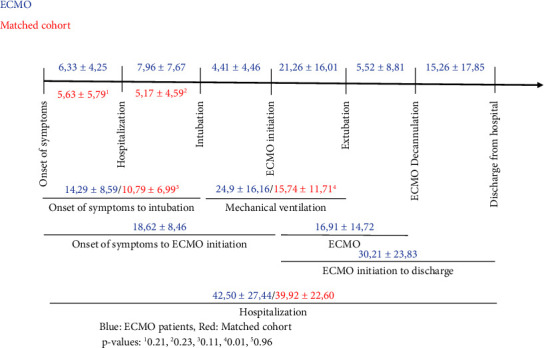
Timeline for mean duration of treatment phases (days).

Figure 3 presents a timeline with the mean duration of treatment phases in the COVID-19 ECMO and matched cohort, and no significant differences were demonstrated in the timeline of onset of symptoms to hospitalization (14.29 ± 8.59 days vs. 10.79 ± 6.99 days, *p*=0.21) and in the timeline of hospitalization to intubation (7.12 ± 6.90 days vs. 5.17 ± 4.59 days, *p*=0.23) between COVID-19 ECMO patients and the matched cohort group. The duration of mechanical ventilation was shorter in the matched cohort compared to the COVID-19 ECMO group (15.74 ± 11.71 vs. 24.9 ± 16.16, *p*=0.01). All other treatment phases were equal in both groups.

In total, 3 COVID-19 ECMO patients (12.5%) suffered from a CVA or stroke. Major bleeding occurred in 17 patients undergoing ECMO treatment (70.83%), and heparin-induced thrombocytopenia was diagnosed in 2 patients after ECMO (8.33%). At last, 5 patients (20.83%) required a second ECMO run of which 3 patients were deceased.

## 4. Discussion

In this propensity-matched cohort study comparing the impact of ECMO and MVA on mortality and complications in severe COVID-19 pneumonia, 24 MVA patients were identified as suitable matches for the 24 ECMO patients in terms of similar sex, P/F-ratio, and date of ICU admission. The analysis also showed that the two groups were not statistically significantly different in terms of age, medical antecedents, clinical frailty score, BMI, and SOFA score on admission. This study demonstrated an almost three-fold risk of ICU mortality (*p* < 0.01) in patients supported with ECMO (45.8%) compared to MVA (16.67%). The difference in three-month mortality between groups was even higher (50% vs. 16.67%; *p*=0.02). Nonetheless, MVA patients were as expected exposed to less lung-protective ventilation settings, including higher peak inspiratory pressures (33.42 ± 8.52 mm; Hg vs. 24.74 ± 4.86 mmHg; *p* < 0.01). Also, maximal PEEP levels (13.52 ± 3.86 mmHg vs. 11.05 ± 2.20 mmHg; *p*=0.01) were higher in the MVA group. ICU-LOS and hospital-LOS were comparable in the ECMO group and the matched cohort group.

These results are not consistent with the findings of the first recently published retrospective propensity-matched cohort study on this topic. Mustafa et al. reported a 3-fold improvement in survival with ECMO, with a mortality rate of 25% with EMCO (*n* = 80) compared to 74% in the MVA cohort (*n* = 80) [9]. In latter study, data were collected from patients treated between March 1st, 2020, and June 9th, 2021 [9].

The first results of ECMO therapy in COVID-19 patients from small Chinese cohorts were discouraging, reporting a very high mortality [24, 25]. A more recently published systematic review including 1896 COVID-19 patients supported with ECMO reported a pooled in-hospital mortality of 37.1% [26]. The variation in study outcomes, however, was rather high with a reported heterogeneity *I*^2^ of 52.8% [26]. This heterogeneity may be explained by differences in the study population, sample size, and/or publication bias. A retrospective analysis of data of the Extracorporeal Life Support Organization Registry and COVID-19 Addendum including 4812 COVID-19 patients receiving ECMO in 2020 across 349 centers within 41 countries showed that in-hospital mortality 90 days after ECMO initiation was between 36.9% and 58.9% [27]. More specifically, early-adopting centers that used ECMO therapy throughout 2020, reported a mortality rate of 36.9% in 1182 patients receiving ECMO on or before May 1st and 51.9% in 2824 patients after May 1^st^ [27]. Late-adopting centers that provided ECMO for COVID-19 only after May 1st, 2020, reported a mortality rate of 58.9% in 806 patients [27]. A large epidemiologic study reporting on hospital mortality in severe COVID-19 patients requiring admission into Belgian ICUs concluded that ECMO therapy is an independent predictor of in-hospital mortality (OR 8.83 (4.50–17.34)) [28]. This reported odds ratio is in line with the odds ratio reported in the present study (5.91 (1.55, 22.58)).

Two large systematic reviews assessing outcomes in COVID-19 patients supported with IMV alone reported overall mortality rates of 43% and 45% and a mortality rate of 36% in the Europe cohort [29, 30]. A large international cohort study comparing the outcome of patients admitted to seven large ICUs in the Euregio Meuse-Rhine, one region across Belgium, The Netherlands, and Germany found the lowest mortality rate in the Belgian subgroup (i.e., 22%, 42%, and 44%, respectively) [3]. The mortality rate in the mechanically ventilated subcohort was 29%, 45%, and 44%, respectively [3].

Thus, it has to be emphasized that compared to the literature, Mustafa et al. reported a very low mortality rate with ECMO and a very high mortality rate with MVA [9]. In contrast, the present study found a mortality rate with ECMO well in the range of values found in the literature but a mortality rate in the matched MVA cohort at the very low end of values reported in the literature. These conflicting results may be explained by a combination of selection bias, intervention bias, and the use of different concomitant treatment strategies. First, despite the application of propensity score matching in both studies, the selection of controls and cases is performed in different ways. In this study, patients were already selected for ECMO candidacy at ICU admission resulting in rather homogeneous control and case groups in terms of age, medical history, and clinical frailty. This selection procedure may also partially explain the low observed mortality rate in the matched cohort group. The mortality rate of the patients not selected for ECMO candidacy in our study was indeed much higher, up to 41.4% in the mechanically ventilated subcohort. In contrast, in the study of Mustafa et al., the selection of controls was performed in a post hoc manner, based on age, ventilatory settings, arterial blood gas results, and presence of severe chronic organ dysfunction or acute multiorgan failure [9]. This selection method may have been associated with a high probability of a selection bias, favoring the use of ECMO in younger patients or those with fewer comorbidities. Second, applied ECMO support strategies also differ significantly between studies. In this study, 23 patients were treated with VV ECMO and only one patient with proven right ventricle (RV) failure with VA ECMO. In contrast, Mustafa et al. utilized single access, dual-stage right atrium to the pulmonary artery cannula resulting in additive right heart support in all patients [9]. However, relatively new and infrequently used, the single access dual-stage right atrium to the pulmonary artery cannula indeed classifies as a right ventricular assist device and has proven benefit for unloading of the failing right ventricle in various clinical settings [31, 32]. This may partially explain the favorable outcomes in the ECMO group of the latter study since numerous studies have demonstrated that COVID-19 infection and ARDS are independent promotors of RV failure [33, 34]. This hypothesis is supported by the findings of another retrospective study that RVAD support at the time of ECMO initiation results in higher in-hospital and 30 days survival versus IMV in specially selected patients with severe COVID-19 ARDS [35]. Another potentially beneficial difference in ECMO strategy in the latter study is the extubation and mobilization of patients while on ECMO [9]. Third, several treatment strategies applied at the JESSA hospital may also explain the low observed mortality rate in the non-ECMO cohort of this study. Early during the first wave, we observed a prevalence of DVT in more than 65% of the intubated and mechanically ventilated COVID-19 patients [20]. Therefore, we implemented a more aggressive thromboprophylaxis protocol including close to therapeutic LMWH dosing, individually tailored with routine anti-Xa measurements and systematically ultrasonography screening for DVT. A before-after study suggested a significant decrease in one-month mortality after the implementation of this more aggressive thromboprophylaxis protocol [11]. It might even be hypothesized that the low mortality rate in the ECMO cohort of Mustafa et al. is partially the result of a more aggressive anticoagulation strategy in this cohort. Conversely, the high mortality rate in the MVA cohort of the latter study might have been partially due to the utilization of a more conservative thromboprophylaxis strategy in this cohort. These hypotheses are supported by the results of a large RCT evaluating the effects of therapeutic LMWH versus standard prophylactic or intermediate-dose heparins for thromboprophylaxis [36]. This RCT concluded that therapeutic LMWH reduces major thromboembolism and death in high-risk hospitalized COVID-19 patients. In contrast, the observation that all patients who died in the ECMO group of the present study experienced major bleeding during their ICU stay may suggest that the applied anticoagulation strategy in this cohort was too aggressive.

Another treatment strategy applied at the JESSA hospital to explain both the low observed mortality rate in the MVA group and the high observed mortality rate in the ECMO group of the present study might be the early adoption of awake-prone positioning. A recently published large multicenter RCT evaluating the efficacy of awake prone positioning concluded that this treatment strategy is safe and has a favorable effect on the primary composite outcome of intubation or death within 28 days of enrolment [37]. However, this randomized controlled trial was not able to detect a statistically significant difference in death between the aforementioned groups 37. It might be hypothesized that awake-prone positioning may have caused exhaustion of the most affected patients in the present study, eventually resulting in a worse outcome for ECMO patients. Finally, in contrast to the present study, Mustafa et al. did not include “date of ICU admission” or “wave” into the propensity score model increasing the likelihood of an asymmetrical distribution of patients across groups over time. An asymmetrical distribution results in an increased risk of both selection bias and treatment bias because patients infected with different virus variants and disease severity are compared to each other and because of changing therapy strategies over time.

Subanalysis showed no association between BMI categories and ICU mortality in both ECMO and MVA groups. In contrast, ICU mortality seems to increase with increasing age (>60 years) with ECMO. These results echo those of the previous studies [38, 39].

This study has several limitations. First, the retrospective single-center design with relatively low numbers of patients negatively impacts the generalizability of our findings. Second, there is a potential impact of the increasing knowledge of pathophysiology and treatment options in COVID-19 over time, which leads to frequent changes in therapeutic strategies, creating a heterogeneous patient population. Third, during the first wave, national Belgian guidelines for admission to ICU and ECMO initiation became more stringent to secure sufficient ICU capacity, also causing heterogeneity in the patient population. Nonetheless, this high threshold for ECMO during the first wave may have enhanced the selection of a well-matched cohort of COVID-19 patients treated with MVA.

In conclusion, we were not able to reproduce the positive results of ECMO therapy in the first propensity-matched cohort study comparing ECMO and MVA in critically ill patients with COVID-19 pneumonia. In contrast, the results of the present propensity-matched cohort study suggest that ECMO therapy may be associated with an up to a three-fold increase in ICU mortality and three-month mortality compared to MVA despite the facilitation of lung-protective ventilation settings. The results of our analysis and the conflicting data in the literature demonstrate the need for randomized controlled multicenter clinical trials on the clinical impact of ECMO therapy in refractory COVID-19-induced ARDS.

## Figures and Tables

**Table 1:** Baseline characteristics.

	COVID-19 ECMO patients (*n* = 24)	COVID-19 non ECMO patients (*n* = 62)	*p* value	Matched cohorts (*n* = 24)	*p*-value
Age	57.66 ± 9.21	58.27 ± 10.19	0.81	61.42 ± 10.02	0.18
Age categories (years)			0.83		0.18
<50	6 (25%)	11 (17.7%)		1 (4.2%)	
51–60	8 (33.3%)	22 (35.5%)		9 (37.5%)	
61–70	9 (37.5%)	24 (38.7%)		11 (45.8%)	
>71	1 (4.2%)	5 (8.1%)		3 (12.5%)	
Gender (male/female)	18 (75%)/6 (25%)	45 (72.60%)/17 (27.40%)	0.82	18 (75%)/6 (25%)	1.00
BMI (kg/m^2^)	33.10 ± 9.16	29.61 ± 5.59	0.23	29.33 ± 5.62	0.15
BMI categories (kg/m^2^)			0.34		0.35
Normal and overweight (18.50–29.99)	13 (54.2%)	38 (61.3%)		14 (58.3%)	
Moderate obesity (30-39.99)	7 (29.2%)	20 (32.3%)		9 (37.5%)	
Severe obesity (>40)	4 (16.7%)	4 (6.4%)		1 (4.2%)	
Rockwood clinical frailty index	2.17 ± 1.20	2.18 ± 0.95	0.57	2.46 ± 1.10	0.39
SOFA score admission	4.42 ± 2.93	3.20 ± 2.11	0.05	4.68 ± 2.66	0.74
Cardiovascular disease	4 (16.70%)	9 (14.50%)	0.80	4 (16.70%)	1.00
Hypertension	9 (37.5%)	21 (33.90%)	0.75	10 (41.70%)	0.77
Diabetes	4 (16.70%)	10 (16.10%)	0.95	4 (16.70%)	1.00
Respiratory disease	2 (8.30%)	10 (16.10%)	0.35	6 (75.0%)	0.08
Malignancy	2 (8.30%)	5 (8.1%)	0.97	3 (12.50%)	0.30
Chronic kidney disease	0 (0.00%)	5 (8.10%)	0.15	2 (8.30%)	0.15
Chronic liver disease	0 (0.00%)	3 (4.80%)	0.27	2 (8.30%)	0.11
Chronic bowel disease	1 (4.20%)	3 (4.80%)	0.89	1 (4.20%)	1.00
Chronic nervous disease	0 (0.00%)	1 (1.60%)	0.53	0 (0.00%)	1.00
Cerebrovascular disease	3 (12.50%)	4 (6.50%)	0.36	2 (8.30%)	0.33
HIV/Aids	0 (0.00%)	0 (0.00%)	1.00	0 (0.00%)	1.00
Hematological disease	1 (4.20%)	2 (3.20%)	0.83	2 (8.30%)	0.28
Obesity	11 (45.80%)	19 (30.60%)	0.18	8 (33.30%)	0.38
Rheumatological disease	2 (8.30%)	3 (4.80%)	0.53	1 (4.20%)	0.55
Dementia	0 (0.00%)	0 (0.00%)	1.00	0 (0.00%)	1.00

Data are expressed as mean ± standard deviation or as frequencies. A *p* value <0.05 is considered statistically significant.

**Table 2:** Progress, complications, and outcomes.

	COVID-19 ECMO patients (*n* = 24)	COVID-19 non ECMO patients (*n* = 62)	*p* value	Matched cohorts (*n* = 24)	*p*-value
LOS ICU (days)	33.21 ± 23.16	17.70 ± 17.56	**<0.001**	30.82 ± 21.48	0.78
LOS total (days)	38.35 ± 25.88	25.43 ± 20.31	**<0.01**	40.86 ± 24.25	0.74
ICU mortality	11 (45.80%)	4 (6.50%)	**<0.001**	4 (16.6%)	**0.02**
3-month mortality	12 (50.00%)	4 (6.50%)	**<0.001**	4 (16.6%)	**<0.001**
Highest SOFA score	12.17 ± 2.46	6.59 ± 4.61	**<0.001**	11.95 ± 4.25	0.54
PaO_2_	62.27 ± 13.38	57.02 ± 12.57	0.12	53.96 ± 11.45	**0.03**
PaCO_2_	49.55 ± 13.16	38.52 ± 11.79	**<0.001**	42.17 ± 11.98	**0.04**
pH	7.32 ± 0.09	7.31 ± 0.89	**<0.001**	7.39 ± 0.19	**0.04**
SaO_2_	89.05 ± 4.56	87.79 ± 5.19	0.47	86.08 ± 5.36	0.08
Lactate	2.05 ± 2.56	1.55 ± 0.54	0.77	1.58 ± 0.65	0.38
P/F ratio	64.59 ± 14.22	72.27 ± 47.26	0.29	59.17 ± 17.82	**0.04**
Invasive mechanical ventilation	24 (100%)	30 (48.39%)	**<0.001**	24 (100%)	1.00
Prone ventilation	24 (100%)	45 (72.60%)	**<0.01**	21 (87.50%)	0.07
PEEP	11.05 ± 2.20	14.48 ± 3.82	**0.03**	13.52 ± 3.86	**0.01**
PIP	24.74 ± 4.86	34.48 ± 7.82	**<0.001**	33.42 ± 8.52	**<0.01**
Neuromuscular blockers	20 (83.33%)	27 (43.55%)	**<0.001**	21 (87.50%)	0.24

*Treatment*					
Corticosteroid	22 (91.7%)	53 (85.5%)	0.44	20 (83.3%)	0.38
Plaquenil	2 (8.3%)	13 (21.0%)	0.16	8 (33.3%)	0.03
Remdesivir	0 (0.0%)	1 (1.6%)	0.53	1 (4.2%)	0.31

*Complications*					
Sepsis	22 (91.70%)	29 (46.80%)	**<0.001**	22 (91.70%)	1.00
CRRT	7 (29.20%)	5 (8.10%)	**0.01**	5 (20.80%)	0.51
AKI	10 (41.70%)	12 (19.40%)	**0.03**	10 (41.70%)	1.00

Data are expressed as mean ± standard deviation or as frequencies. A *p*-value <0.05 is considered statistically significant.

## Data Availability

Due to the applicable privacy regulation (GDPR) and Good Clinical Practices (GCPs) legislation, the full underlying dataset supporting the study cannot be provided. This dataset contains potentially identifying information, for example, age, BMI, and comorbidities such as diabetes mellitus leading to a unique subject in the dataset. Therefore, descriptive statistics have been used for a general overview of our study population, and all other relevant information is provided in Table 1. Anonymized data are available on motivated request and can be sent to Prof. Dr. Björn Stessel; Stadsomvaart 11; 3500 Hasselt, Belgium; bjorn.stessel@jessazh.be; AND Jessa Ziekenhuis, Data Protection Officer (DPO); Stadsomvaart 11; 3500 Hasselt, Belgium; DPO@jessazh.be.

## Abbreviations

ARDS: Acute respiratory distress syndrome
MVA: Maximum ventilation alone
DVT: Deep vein thrombosis
DNI: Do-not-intubate
VTE: Venous thromboembolism
IMV: Invasive mechanical ventilation
ICU: Intensive care unit
LMWH: Low molecular weight heparine
UFH: Unfractionated heparine
aPTT: Thromboplastin time aPTT
INR: International normalized ratio
NMBA: Neuromuscular blocking agents
PEEP: Positive end expiratory pressure
P/F ratio: Ratio of arterial oxygen partial pressure to fractional inspired oxygen concentration
SARS-CoV-2: Severe acute respiratory syndrome coronavirus 2
SD: Standard deviation.

## Supplementary Materials

Supplementary Materials Table 1: distribution of COVID-19 patients stratified by the date of inclusion. Table 2: baseline characteristics of the COVID-19 ICU AA group (*n* = 129). Table 3: outcomes of the COVID-19 AA group (*n* = 129). Table 4: complications and cause of death COVID-19 ECMO patients (*n* = 24).
