# Supplementary material for: Extracorporeal Membrane Oxygenation to Support COVID-19 Patients: A Propensity-Matched Cohort Study
Source: Crit Care Res Pract. 2023 Jun 12;2023:5101456. doi: 10.1155/2023/5101456 (PMC10279486; doi:10.1155/2023/5101456)
Supplement: Supplementary Materials — Table 1: distribution of COVID-19 patients stratified by the date of inclusion. Table 2: baseline characteristics of the COVID-19 ICU AA group (n = 129). Table 3: outcomes of the COVID-19 AA group (n = 129). Table 4: complications and cause of death COVID-19 ECMO patients (n = 24). [file 5101456.f1.docx]

Supplementary files

Table 1. Distribution of COVID-19 patients stratified by date of inclusion.

|  | COVID-19 ECMO patients (n=24) | COVID-19 AAA patients (n=62) | COVID-19 matched patients (n=24) |
| --- | --- | --- | --- |
| **Wave 1**  (March 2020- August 2020) | 3 | 14 | 5 |
| **Wave 2**  (September 2020- December 2020) | 9 | 20 | 7 |
| **Wave 3** (January 2021- April 2021) | 10 | 20 | 9 |
| **Wave 4** (May 2021- October 2021) | 2 | 8 | 3 |

Wave 1 and 2 in Belgium were caused by the D614G variant, wave 3 by the alpha variant and wave 4 by the delta variant.

Table 2. Baseline characteristics of COVID-19 ICU AA group (n=129)

|  | COVID-19 AA patients (n=129) |
| --- | --- |
| Age (years) | 70.53 ± 8.84 |
| Age categories (years)  <50  51-60  61-70  >71 | 3 (2.3%)  14 (10.8%)  38 (29.4%)  74 (57.3%) |
| Gender (males/females) | 85 (65.9%) /44 (34.1%) |
| BMI (kg/m2) | 28.22 ± 5.71 |
| BMI categories (kg/m2)  Normal and overweight (18.50-29.99)  Moderate obesity (30.00-39.99)  Severe obesity (>40)  Missing data | 93 (72.1%)  29 (22.5%)  4 (3.1%)  3 (2.3%) |
| Rockwood Clinical Frailty Index | 3.51 ± 1.44 |
| SOFA score at admission | 4.84 ± 3.05 |
| Cardiovascular disease | 46 (35.9%) |
| Hypertension | 75 (58.6%) |
| Diabetes | 44 (34.1%) |
| Respiratory disease | 21 (16.4%) |
| Malignancy | 11 (8.6%) |
| Chronic kidney disease | 14 (10.9%) |
| Chronic liver disease | 5 (3.9%) |
| Chronic bowel disease | 7 (5.5%) |
| Chronic nervous disease | 1 (0.8%) |
| Cerebrovascular disease | 19 (14.8%) |
| HIV/AIDS | 1 (0.8%) |
| Hematological disease | 5 (3.9%) |
| Obesity | 6 (4.7%) |
| Rheumatological disease | 38 (29.7%) |
| Dementia | 1 (0.8%) |

Data are expressed as mean ± SD or as frequencies (%).

Table 3. Outcomes of COVID-19 AA group (n=129)

| Outcomes | COVID-19 AA patients (n=129) |
| --- | --- |
| Invasive mechanical ventilation | 82 (63.6%) |
| Mortality | 37 (28.7%) |

Data are expressed as frequencies (%).

Table 4 . Complications and cause of death COVID-19 ECMO patients (n=24)

|  | COVID-19 ECMO patients (n=24) |
| --- | --- |
| CVA   - Ischemic stroke - Intracranial bleeding | 3 (12.5%)  2 (8.3%)  1 (4.2%) |
| HIT | 2 (8.3%) |
| Heart failure | 10 (41.7%) |
| Bacterial co-infection | 20 |
| Fungal co-infection | 2 (8.3%) |
| VTE   - DVT - LE | 15 (62.5%)  14 (58.3%)  1 (4.2%) |
| Mortality   - Multi organ failure - Ischemic stroke - Intracranial bleeding - Disseminated fungal infection - Dislocation canula in subclavia - Major lung bleeding | 11 (45.8%)  6 (25.0%)  1 (4.2%)  1 (4.2%)  1 (4.2%)  1 (4.2%)  1 (4.2%) |

Data are expressed as frequencies (%).
